# Prevalence of symptoms of temporomandibular disorders, oral behaviors, anxiety, and depression in Dentistry students during the period of social isolation due to COVID-19

**DOI:** 10.1590/1678-7757-2020-0445

**Published:** 2020-11-30

**Authors:** Rodrigo Antonio De Medeiros, Danielle Leal Vieira, Emily Vivianne Freitas Da Silva, LilianA Vicente Melo De Lucas Rezende, Rodrigo Wendel Dos Santos, Lucas Fernando Tabata

**Affiliations:** 1 Universidade de Brasília Faculdade de Ciências da Saúde Departamento de Odontologia BrasíliaDistrito Federal Brasil Universidade de Brasília, Faculdade de Ciências da Saúde, Departamento de Odontologia, Brasília, Distrito Federal, Brasil.; 2 Universidade Estadual Paulista Faculdade de Odontologia de Araçatuba Departamento de Materiais Odontológicos e Prótese AraçatubaSão Paulo Brasil Universidade Estadual Paulista, Faculdade de Odontologia de Araçatuba, Departamento de Materiais Odontológicos e Prótese, Araçatuba, São Paulo, Brasil.; 3 Dentista em clínica privada BrasíliaDistrito Federal Brasil Dentista em clínica privada, Brasília, Distrito Federal, Brasil.

**Keywords:** Temporomandibular Joint Dysfunction Syndrome, Anxiety, Anxiety disorders, Myalgia, COVID-19

## Abstract

Temporomandibular dysfunction (TMD), anxiety, and depression are disorders that, due to the current lifestyle, are affecting an increasing portion of the population. Investigating the prevalence of the symptoms of these disorders during the quarantine due to the coronavirus 2019 pandemic (COVID-19) is important to outline clinical strategies for patient care.

**Objective:**

This study assessed the prevalence of TMD symptoms, anxiety, depression, and oral behaviors and their associations during the social isolation due to COVID-19.

**Methodology:**

Questionnaires were used to assess TMD symptoms in accordance with the Diagnostic Criteria for Temporomandibular Disorders: clinical protocol and assessment instruments, a questionnaire to verify oral behaviors and Hospital Anxiety and Depression Scale to assess symptoms of anxiety and depression in students of dentistry at the Faculty of Health Sciences of the University of Brasília in May 2020. Qualitative data were subjected to descriptive statistics and chi-squared analysis (p<0.05). The relationship between quantitative and qualitative data was evaluated using Spearman's rho correlation (p<0.05).

**Results:**

There was a high prevalence of TMD symptoms, anxiety, and depression in the participants, resulting in association between gender and anxiety symptoms (p=0.029). There was a positive correlation between oral behaviors and TMD symptoms (r=0.364; p<0.001), between oral behaviors and anxiety symptoms (r=0.312; p=0.001), and between oral behaviors and symptoms of depression (r=0.216; p=0.021).

**Conclusion:**

Social isolation due to the COVID-19 pandemic has an impact on the prevalence of TMD symptoms, anxiety, and depression.

## Introduction

COVID-19 (coronavirus disease 2019) quickly spread in late January 2020 and attracted enormous attention worldwide.^1^ The new disease caused by the novel coronavirus (SARS-CoV-2) originated from a market in Wuhan, China, in early December 2019.^2^ On January 7, 2020, the Chinese Center for Disease Control and Prevention identified and isolated this new coronavirus, calling it severe acute respiratory syndrome coronavirus 2 (SARS-CoV-2). SARS-CoV-2 can be transmitted from one person to another through close contact, leading to COVID-19.^3^ Infected patients can develop severe respiratory distress, requiring intensive care, and can be fatal.^1^^,^^3^ Owing to this scenario, several countries in the world are adopting restrictive quarantine measures to control the spread of the virus and the collapse of health systems.

It is not uncommon for people with a confirmed diagnosis or with suspected COVID-19 to experience great psychological pressure. Health professionals are also susceptible to these problems, as they must care for infected patients, to decrease or, in some cases, to restrict living with their family, among other factors.^4^ In addition, people who are quarantined, fulfilling social isolation, restricted to leave, concerned about infection, afraid of death, lack information, and who have lost daily social relationships, can further experience high levels of anxiety and depression.^5^

Psychological factors are associated with the development of some diseases and disorders, including temporomandibular disorders (TMD), which is a collective term used to describe disorders related to temporomandibular joints (TMJs) and masticatory muscles, which are primarily responsible for the movement of TMJs and related structures.^6^ The etiology of TMD is multifactorial, including the combined action of environmental, biological, psychological, biomechanical, and neuromuscular factors.^6^^,^^7^ Symptoms are generally jaw pain, ear pain, toothache (of non-dental origin), joint pain, headache, and mandibular functional limitation.^8^^,^^9^ More than 50% of the population present some level of TMD, but only 3.6% to 7% require treatment.^10^ The highest prevalence is in women aged 20 to 40 years.^10^

Currently, the bio-psychosocial model studies the etiology of TMD due to biological factors, such as genetic or biochemical factors, psychological factors, such as anxiety, stress, and depression, and social factors, such as culture, family behavior, and socioeconomic status. However, the mechanisms by which psychological factors influence the development of TMD remain unknown.^7^

Therefore, studies that assess the influence of the COVID-19 pandemic, and its consequences on TMDs are important. Thus, the objective of this study was to assess the prevalence of TMD symptoms, anxiety, depression, and oral behaviors and their associations during the social isolation due to COVID-19. The null hypothesis is that there will be no association between TMD symptoms, anxiety, depression, and oral behaviors.

## Methodology

This was a cross-sectional study performed on dental medicine students from the School of Health Sciences of the University of Brasília (FS/UnB). The study was approved by the National Council of Ethics in Research (CAAE: 30637620.2.0000.0008).

A minimum sample size of 104 participants was determined based on the size of the student population of this course of 220 students, an accuracy of 5%, an estimated prevalence of 15%, and a 95% confidence interval, using an online sample size calculator. (http://sampsize.sourceforge.net/iface). All students of both genders were invited to participate in the research. All subjects signed an informed consent form before participation. Students on medication for anxiety and/or depression were excluded.

Due to the impossibility of clinical evaluation because of the social isolation due to COVID-19, the evaluation of TMD symptoms, oral behaviors, anxiety, and depression was carried out through questionnaires, applied between May 12 and 19, 2020, and made available through a QR code and link, which was sent to class e-mails and disseminated on social networks.

General data such as name, age, gender, and medication use were collected. In addition, the following was asked:

How is your isolation going? Total social isolation - without ever leaving home; Partial social isolation - at home, but going to the market, pharmacy, and other essential services, or; No isolation - I am not performing social isolation.

Are you carrying out any physical activity at home? Yes or no.

### Assessment of the presence of TMD symptoms

The Diagnostic Criteria for Temporomandibular Disorders: clinical protocol and assessment instruments (DC/TMD) questionnaire^11^ was translated and validated into Brazilian Portuguese and self-administered with objective questions about TMD symptoms. The answered questionnaires were analyzed by a specialist in the field and the participants were categorized into three groups according to their symptoms in the last 30 days: without TMD, with non-painful TMD, or painful TMD. For this, they were classified as either having painful TMD, based on the first two questions of the questionnaire related to the presence of pain and headache, or having non-painful TMD, based on questions related to the presence of joint sounds, closed locking, and/or open locking.

### Assessment of the presence of parafunctional habits/oral behaviors

The DC/TMD oral behavior checklist (OBC) questionnaire,^11^ translated into and validated for Brazilian Portuguese, was used for self-completion, containing objective questions about the frequency of oral behaviors during sleep and during waking. For each OBC item, the participant was asked to report the frequency of occurrence in the last 30 days, using the options “none of the times” (0), “a small part of the time” (1), “some part of the time” (2), “most of the time” (3), “all the time” (4) for activities during awake hours, and “none of the time” (0), “< 1 night/month” (1), “1-3 nights/month” (2), “1-3 nights/week” (3), “4-7” nights/week” (4) for activities during sleeping hours. The values were added and assigned to each participant, where the higher the value, the more oral behaviors the individual would present.

### Assessment of anxiety and depression

The Hospital Anxiety and Depression Scale (HADS) was used to assess symptoms of anxiety and depression. It is made of 14 items, 7 of which assess anxiety (HADS-A) and 7 depression (HADS-D).^12^ Each of its items can be scored from 0 to 3, making up a maximum score of 21 points for each scale.^13^

Responses to the HADS items were obtained to assess frequency of anxiety and depression. The recommended cut-off points were adopted for both subscales:^14^

HAD-anxiety: no anxiety: 0 to 7; with anxiety: ≥ 8.

HAD-depression: no depression: 0 to 7; with depression: ≥ 8.

### Data analysis

The results were tabulated and subjected to statistical analysis. Statistical analysis was performed using SPSS software (version 24.0, SPSS Inc., Chicago, USA). Descriptive statistics were used to analyze the demographic data of the participants included in the study. The normality analysis was performed using the Shapiro-Wilk test, with no normality for OBC values. Spearman's rho correlation was performed to verify the relationship between OBC values and TMD symptoms, anxiety symptoms, and depression symptoms. The Chi-Squared analysis was performed to verify the association between sex and TMD symptoms, gender and anxiety symptoms, gender and depression symptoms, isolation and TMD symptoms, isolation and anxiety symptoms, isolation and depression symptoms, physical activity and TMD symptoms, physical activity and anxiety symptoms, physical activity and depression symptoms, TMD symptoms and anxiety symptoms, and TMD symptoms and depression symptoms. All analyses were performed with a 5% level of significance.

## Results

A total of 147 students started the process of filling the questionnaires, 127 of whom completed the questionnaire. Fourteen students were excluded, leaving 113 valid questionnaires. Of the total participants, 87 were female (77%) and 26 were male (23%), with a mean age of 21.46 ± 2.37 years.

Of the 113 participants, 87 practiced partial social isolation (77%), 24 practiced total social isolation (21.2%), and 2 did not practice social isolation (1.8%). Regarding physical activity, 54 participants did not perform activities (47.8%) and 59 participants exercised at home (52.2%).

A total of 51 participants had no symptoms of TMD in the last 30 days (45.2%), 31 had symptoms of non-painful TMD (27.4%), and 31 had symptoms of painful TMD (27.4%). Fifty-seven participants had no anxiety symptoms, (50.4%), of which 56 had anxiety symptoms (49.6%) in the last 30 days. Four participants received TMD treatment before the COVID-19 pandemic, of which 3 had symptoms of painful TMD in the last 30 days and 1 had no TMD symptom. Sixty-nine participants had no depression symptoms (61.1%), while 44 had depression symptoms in the last 30 days (38.9%).

No association was found between TMD symptomatology and gender, anxiety symptoms, depression symptoms, type of social isolation, and physical activity (Table 1). The association between anxiety symptoms and gender was verified [X2 (2) = 4,769; p=0.029]; however, there was no association between anxiety symptoms and type of social isolation and physical activity (Table 2). There was no association between depression symptoms and gender, type of social isolation, or physical activity (Table 3).

**Table 1:** Frequency of study participants regarding the association between TMD symptoms and different analysis variables (Chi-squared, p<0.05)

Variables		No TMD	Non-painful TMD	Painful TMD	P-value
Gender	Male	10	11	5	0.143
	Female	41	20	26	
Anxiety	With symptoms	22	15	19	0.277
	Without symptoms	29	16	12	
Depression	With symptoms	18	13	13	0.771
	Without symptoms	33	18	18	
Isolation	Parcial isolation	39	24	24	0.758
	Full isolation	12	6	6	
	Nol isolation	0	1	1	
Physical activity	Yes	31	13	15	0.224
	No	20	18	16	

**Table 2:** Frequency of study participants regarding the association between anxiety symptoms and different analysis variables (Chi-squared, p<0.05)

Variables		With anxiety symptoms	Without anxiety symptoms	P-value
Gender	Male	8	18	0.029*
	Female	48	39	
Isolation	Parcial isolation	39	48	0.166
	Full isolation	16	8	
	Nol isolation	1	1	
Physical activity	Yes	25	34	0.110
	No	31	23	

**Table 3:** Frequency of study participants regarding the association between the symptoms of depression and different analysis variables (Chi-squared, p<0.05)

Variables		With depression symptoms	Without depression symptoms	P-value
Gender	Male	8	18	0.330
	Female	36	51	
Isolation	Parcial isolation	32	55	0.686
	Full isolation	11	13	
	Nol isolation	1	1	
Physical activity	Yes	20	39	0.251
	No	24	30	

There was a positive correlation between oral behaviors (using the OBC) and TMD symptoms (ρ=0.364 and p<0.001), between oral behaviors and anxiety symptoms (ρ=0.312 and p=0.001), and between oral behaviors and symptoms of depression (ρ=0.216 and p=0.021) (Table 4).

**Table 4:** Spearman's rho correlation between oral behavior value (OBC) and TMD symptoms, anxiety symptoms and depression symptoms (p<0.05)

Spearman's rho correlation	OBC
TMD symptoms	Correlation coefficient = 0.364
	p<0.001*
Anxiety symptoms	Correlation coefficient = 0.312
	p=0.001*
Depression symptoms	Correlation coefficient = 0.216
	p=0.021*

## Discussion

The null hypothesis of the study was partially accepted since there was an association between gender and symptoms of anxiety and a correlation between oral behaviors and symptoms of TMD, anxiety, and depression. The first case of COVID-19 in Brazil was confirmed on February 26, in the city of São Paulo. With the emergence of new cases, Brasília, located in the Federal District and capital of Brazil, had its first confirmed case on March 5, 2020, and there was a government decree demanding the interruption of classes and commercial activities from March 12, 2020, whereas on March 13 it was decided that classes should stop and academic calendar be suspended, starting on March 23. The dental medicine students of the University of Brasilia (UnB) started academic activities in 2020, and the academic calendar remains suspended until the present moment (May 30, 2020). This study was carried out 68 days after the decision to suspend academic activities, and all responses were influenced by the period of social isolation.

The results demonstrated a high rate of symptoms of anxiety and depression, at 49.6% and 38.9%, respectively. A recent study carried out in China showed that 12.9% of the population affected by the quarantine showed symptoms of anxiety and 22.4% of depression.^15^ A recent review showed negative psychological effects, including post-traumatic stress, confusion, and anger, as a consequence of the longer quarantine duration, fear of infection, frustration, boredom, inadequate information, financial loss, restricted movement, and the loss of habitual routine and social contact have also been reported.^5^ In addition, regarding university students, there was a change in life and work planning due to the pandemic, with the possibility of graduation delay and uncertainties of the academic future.^16^ A recent study evaluated 44 447 Chinese university students and found a prevalence of 7.7% of anxiety symptoms and 12.2% of depression symptoms.^17^ Another study performed on Spanish students showed rates of 34.2% for moderate to severe depression symptoms and 21.3% of anxiety symptoms.^18^ Due to this divergence of data, the influence of the pandemic on the psychological factors of university students is not yet known. Considering that the UnB adopted some measures of psychosocial help for students and civil servants, providing psychological assistance online and some extension projects of the School of Health Sciences were reformulated to help the academic and general population in the period of isolation. However, there is a need for wider dissemination of these services and the continuity of psychological assistance after the COVID-19 pandemic.

In the present study, there was a statistically significant association between gender and symptoms of anxiety, with 55.2% of women and 30.8% of men showing symptoms of anxiety. Anxiety symptoms are more common in women, especially among adolescents.^19^ Studies have shown that women have a higher prevalence, of psychiatric disorders related to stress throughout life, such as depression and anxiety disorders, while men have a higher prevalence of externalizing disorders, for example, aggression.^20^ This suggests that men and women may have different neural resources in response to stress-related anxiety.^21^ A recent study showed higher levels of anxiety, depression, and health anxiety in women during the COVID-19 pandemic.^22^ Anxiety disorder has been reported to be three times higher in women than in men during the pandemic.^23^ In addition, females were more potent predictors of post-traumatic stress disorder symptoms after pandemics.^24^

In this study, there was no statistically significant association between anxiety or depression symptoms and TMD symptoms. However, this association has been demonstrated in the literature, and anxiety is more associated with muscle pain and joint pain, as well as with depression.^25^ Depression and anxiety can contribute to TMD, interacting with pain-modulating networks, lowering the threshold, or altering the perception of pain in patients suffering from anxiety or depression, although the exact mechanism remains unclear.^26^ It is possible that if the sample was larger and encompassed other population profiles, such as health professionals who are working directly with the treatment of patients with COVID-19, and patients who are part of the risk groups, there will possibly be an association between TMD symptoms, mostly painful TMD and symptoms of anxiety and depression. However, more studies must be conducted to better understand this relationship during the period of COVID-19. It is known that the prevalence of TMD in university students is high according to data confirmed in several studies prior to the pandemic,^27^^–^^29^ and there are still no studies evaluating the relationship between anxiety and depression symptoms and TMD symptoms during the COVID-19 pandemic. Therefore, the results of the present study must be interpreted with caution. In addition, it is important to continue monitoring the research participants after the end of the pandemic, as according to Almeida-Leite, Stuginski-Barbosa and Conti^30^ (2020), the occurrence of TMD signs and symptoms after the pandemic is expected to follow a similar pattern to post-traumatic stress syndrome.

This study showed a positive association between oral behaviors and symptoms of TMD, anxiety, and depression. Chow and Cioffi^31^ (2019) reported an association between oral behaviors during wakefulness (clenching teeth, leaving the jaw in a rigid position, pressing the tongue against the teeth, playing with the tongue, cheeks, lips, or chewing gum) and painful TMD. These habits are more frequent in patients with painful TMD and are activities that require repeated and sustained contraction of the muscles of the jaw, which can result in muscle overload, local ischemia, and pain.^32^ The frequency of oral behaviors is increased in patients with greater anxiety.^33^ People with high levels of anxiety were shown to have a higher frequency of oral behaviors if pain was present.^34^ Moreover, pain increased the somatic body sensation and contributed to hypervigilance.^31^ Studies evaluating oral conditions with symptoms of TMD, anxiety, and depression during the COVID-19 pandemic have not been found in the literature.

The present study showed a low percentage of total social isolation (21.2%). Possibly, this result may be associated with the profile of the public analyzed and the quarantine time. Previous studies show that high-risk patients are over 70 years old and have preexisting health problems, such as respiratory diseases, cardiovascular diseases, cancer,^35^ hypertension, diabetes, smoking patients, chronic obstructive pulmonary disease, and kidney diseases.^36^ The lower risk of complications may have resulted in relaxation concerning social isolation, with most students reporting that they were experiencing partial social isolation, leaving home at some point during the week. Another factor that may have influenced this result is the number of days of social isolation since monitoring vehicles show that the index of social isolation, over the days, has decreased. The Federal District had a rate of 65.6% on March 22, 2020, and currently (on May 30, 2020), it stands at 39.2%.^37^ However, it is essential to emphasize the importance of government measures to reduce the spread of the SARS-CoV-2 virus. A study carried out in the United States of America showed that if there were no social distance measures, the COVID-19 growth rate could have been ten times higher.^38^

Thus, investigations about the influence of the pandemic and its consequences on TMDs should be carried out to better understand the current situation, and how it will influence the post-pandemic in orofacial pain. The major limitation of the present study was that it was carried out in a very specific population. Therefore, the results cannot be extrapolated convincingly to the general population.

## Conclusion

Social isolation and stressful situations due to the COVID-19 pandemic can increase the number of people with symptoms of TMD, anxiety, and depression. Further studies should be carried out during and after the social isolation period to assess the consequences of the pandemic on psychological alterations and TMDs.
